# A rare case of extensive neurogenic heterotopic ossification: a case report

**DOI:** 10.1186/s12891-024-07369-2

**Published:** 2024-04-23

**Authors:** Vasav Somani, Ashraf Shaikh, Mohan. M. Desai, Rajan Gupta

**Affiliations:** Department of Orthopaedics, Seth G.S.M.C & K.E.M.H., Mumbai, Parel, 400012 India

**Keywords:** Neurogenic heterotopic ossification, Traumatic brain injury, Spinal cord injury, Spasticity

## Abstract

**Introduction:**

Neurogenic Heterotopic ossification (NHO) is a potential sequalae and a detrimental complication following neurological insult. It is characterized by formation of localized gradually progressive, peri-articular lamellar bone formation in extra-skeletal tissues. We would like to report a rare case of heterotopic ossification involving all 4 limbs, in which we tried to restore joint mobility to improve his functional status so that he could perform his daily tasks.

**Case presentation:**

We present a case of a 33-year-old bed ridden male, diagnosed with NHO involving all 4 limbs (bilateral hip, right knee, right shoulder, left elbow). The patient had a crippled posture, significant pain and impaired range of motion hampering movement of all four limbs which prevented him from lying down supine, sitting, walking and performing activities of daily living. After three surgeries, the patient achieved wheelchair mobilization and upright posture with the assistance of calipers.

**Conclusion:**

The management of NHO requires a multidisciplinary approach involving orthopaedic surgeons, neurologists & rehabilitation specialists. Prognosis of NHO depends on factors such as extent of ossification, underlying neurological condition & patients overall health.

## Background

Maiden description of heterotopic ossification after neurological injury was by Dejerne & Ceiller in soldiers who had suffered spinal injury in World War I [1]. Heterotopic ossification after traumatic brain injury was first described by Roberts, who described elbow involvement in patients with brain injury. Recent studies show that the prevalence of NHO in patients with traumatic brain injury is 10–20%, and 10% of these patients develop severe limitation of joint motion [1, 2]. NHO typically occurs within 2 months of the CNS insult and is fully developed by the end of 2 years [3]. The presentation is broad and can range from pain to limited motion to complete ankylosis. NHO involving 2–3 major joints is common, but no case involving 5 major joints has been described in the literature to the best of our knowledge.

Written informed consent was obtained from the patient for publication of this case report.

## Case history

**Fig. 1:**
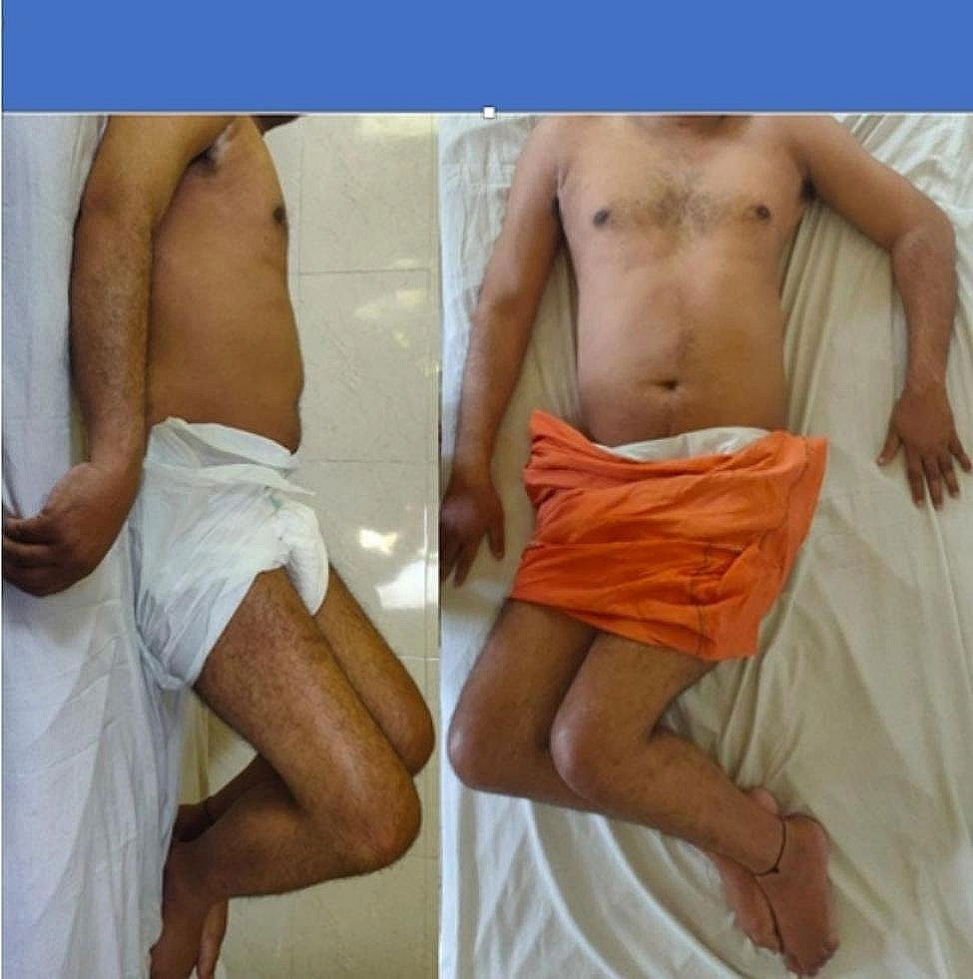
Clinical presentation

A 33-year-old male, bank manager by profession, presented to our out-patient department on a stretcher with complaints of restricted movement and deformity in bilateral hip, right knee, right shoulder & left elbow since two years (Fig. 1). He was involved in a road traffic accident two years back due to which he suffered a traumatic brain injury. A computed tomography scan done for the same diagnosed subdural hemorrhage for which a surgery in the form of coronal burr-holes was performed. The patient was in a comatose state for 10 days post-surgery and was been bed-ridden since then. An MRI scan done post injury was suggestive of diffuse axonal injury.

On neurological examination, patient has signs of upper motor neuron lesion (Spasticity, clonus, Babinski, DTR Exaggerated) with poor hand grip.

On examination of his lower limbs, the following findings were observed. (Table 1)

**Table 1:** Detail of deformity & Range of Motion of all the joints

Right	Attitude of Limb	Left
45 degrees Flexion	Hip Ankylosed	45 degrees Flexion
20 degree Abduction	Coronal plane deformity of hip	20 degree Adduction deformity
90 degrees Flexion	Knee (Deformity)	90 degrees flexion deformity
Ankylosed in 10 degree abduction	Shoulder	Normal
Normal	Elbow	Ankylosed in 45 degrees flexion

Our goal was to achieve wheelchair mobilization for the patient and correction of deformities to ensure personal hygiene.

**Fig. 2:**
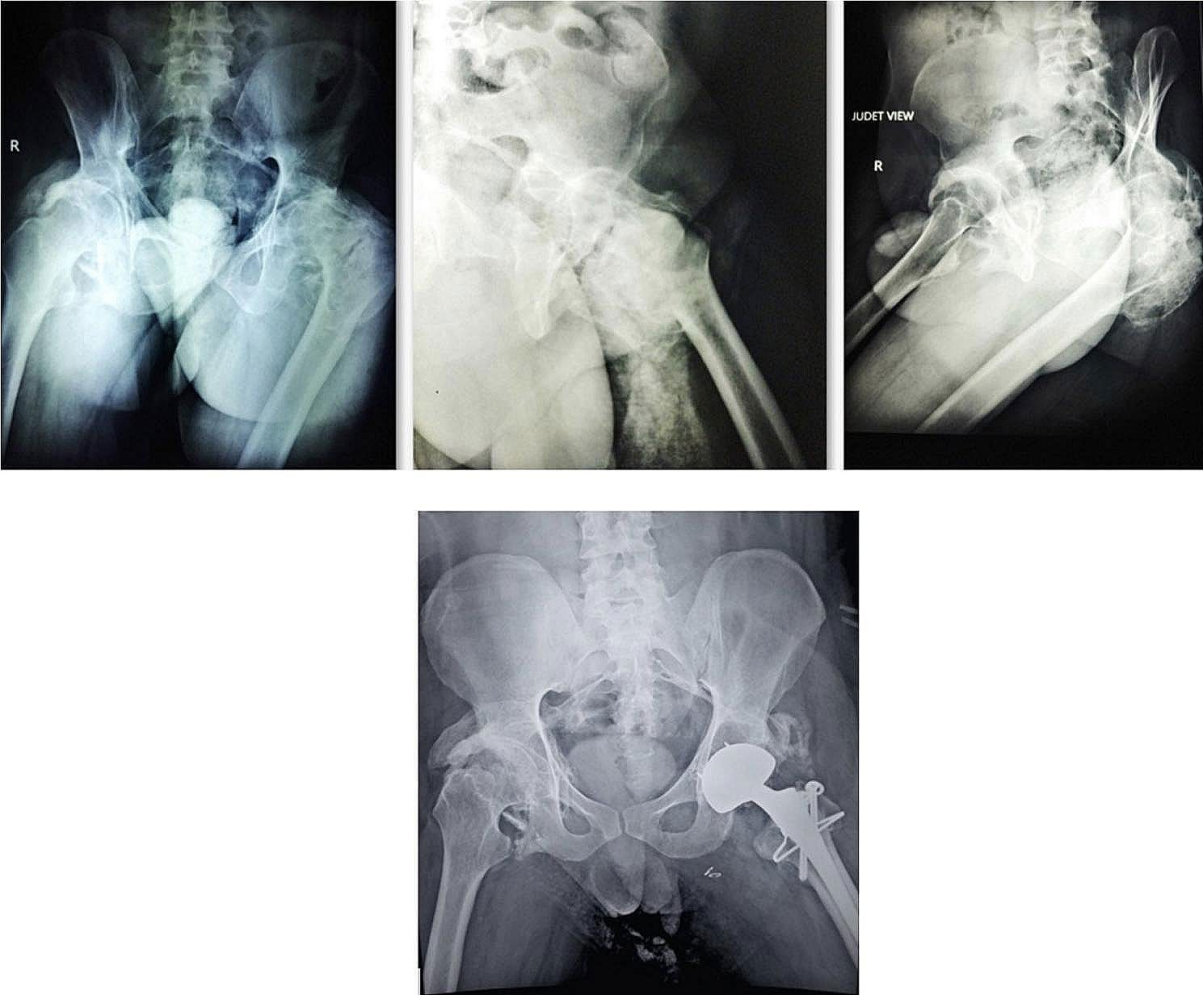
Heterotopic Ossification around Hip

Left hip was significantly affected by HO and therefore was tackled first. Total Hip Arthroplasty was performed along with excision of the HO. Identification of bony landmarks was extremely difficult and judicious use of the C-arm was made. The Superior Gluteal nerve had to be protected, therefore the HO excision in the abductors was carried out with utmost care. We used a dual mobility bearing to reduce the chances of dislocation post-operatively (Fig. 2). Complete correction of coronal & saggital plane deformity had been obtained.

**Fig. 3:**
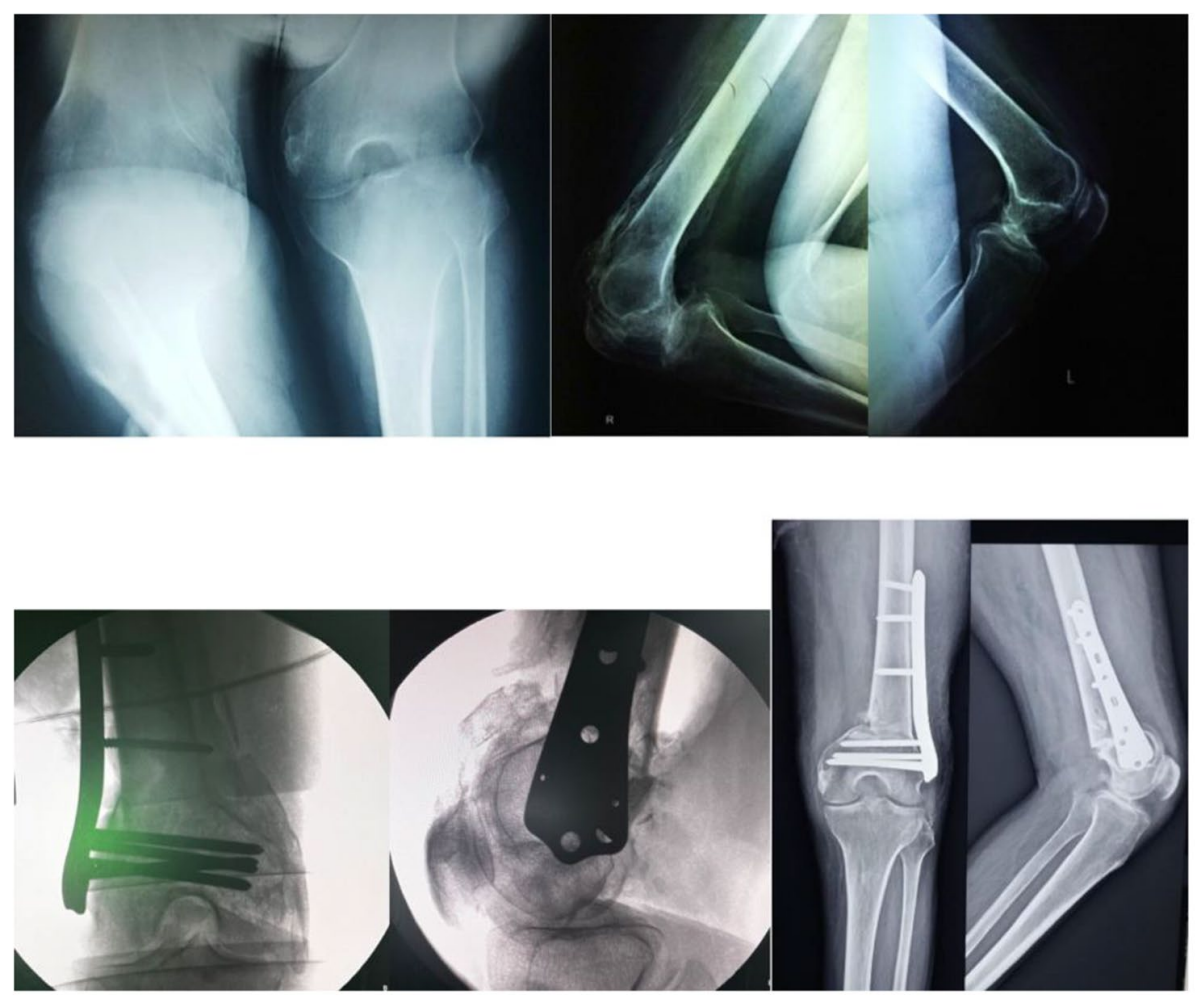
(Row: R → L) a. HO around Right & Left Knee (Antero-posterior & Lateral radiographs of Right & Left Knee) b. Post-operative radiographs after HO Excision & Deformity correction (Antero-posterior & Lateral radiographs of Right & Left Knee)

**Fig. 4:**
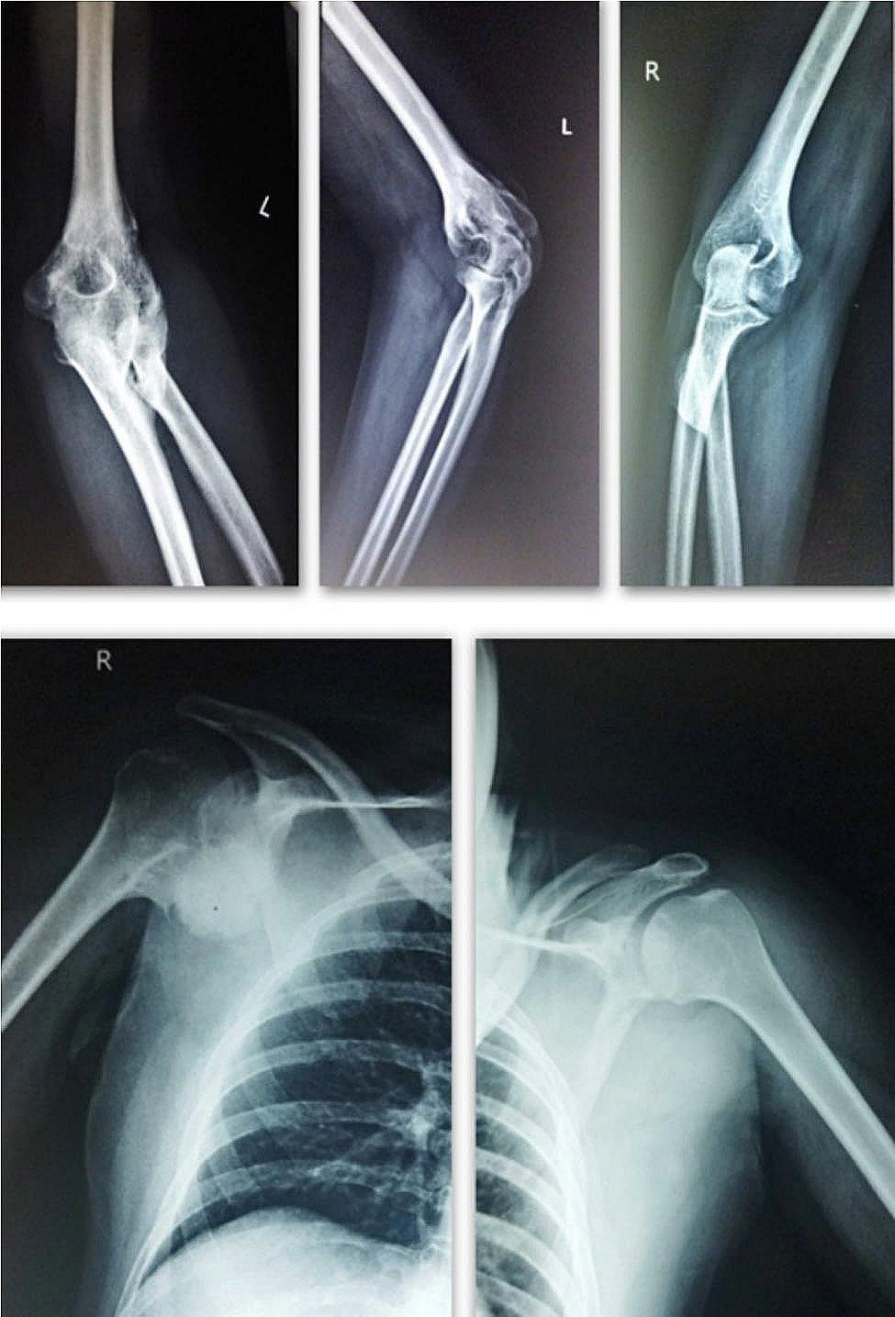
Heterotopic Ossification around Right shoulder and Left elbow

**Fig. 5:**
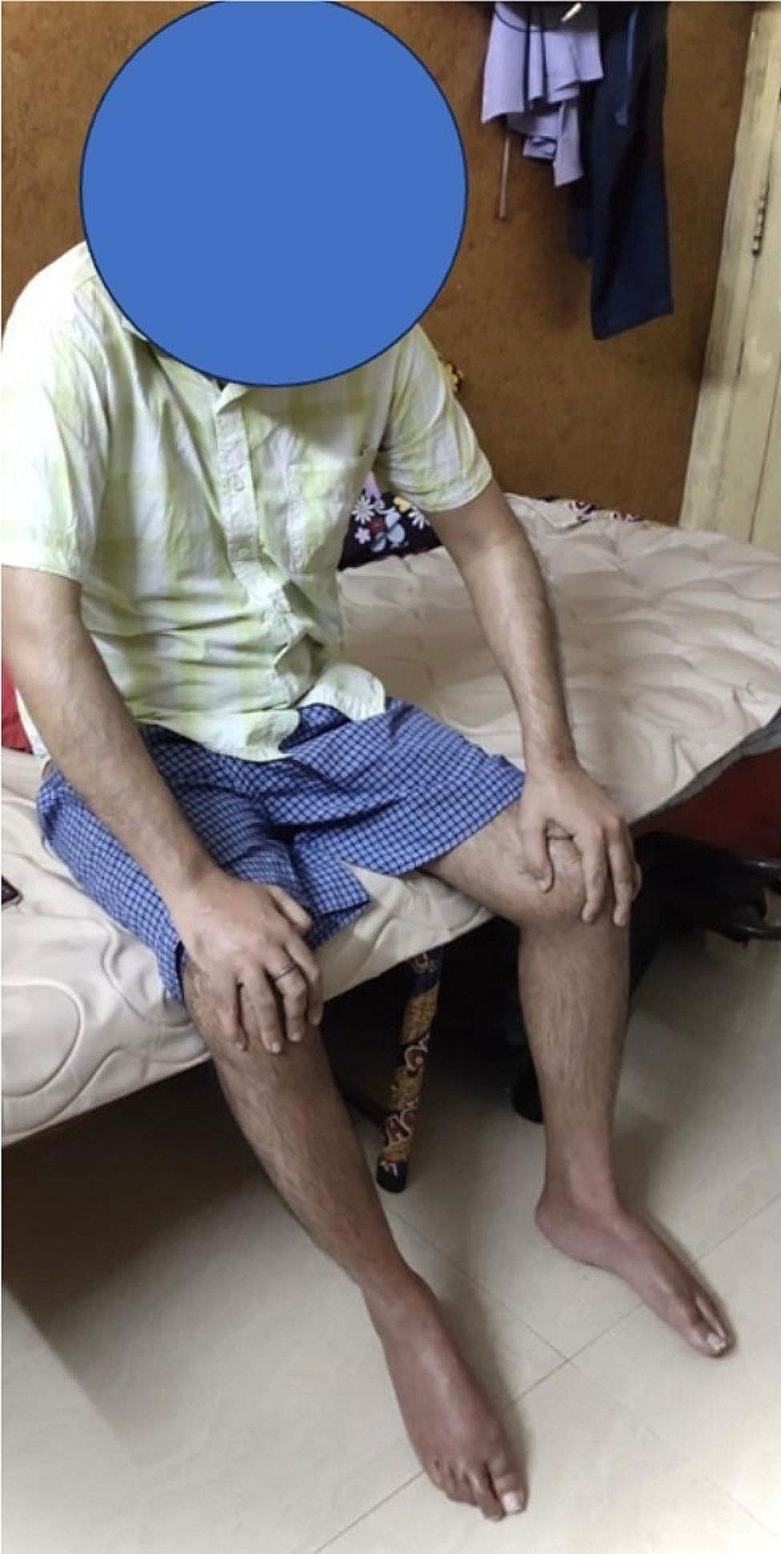
Clinical Image at 2 years follow up. Patient is mobilized with able to sit independetly

The right and the left knee flexion deformities were tackled with hamstring muscles tenotomies and deflexion osteotomies. After general anesthesia, the knee deformities were corrected by 20 degrees due to muscle relaxation. Hamstring tenotomy further corrected the deformity by 25 degrees. This was followed by a lateral approach to the distal femur with extension till the knee joint (Swashbuckler) approach. The HO surrounding the knee joint interfering with the movement was excised, the quadriceps muscles were separated from the HO tissue. An anterior closing wedge osteotomy was then performed in the form of a trapezoidal wedge. The decision to take out a trapezoidal wedge instead of a triangular one was taken as complete correction of such severe flexion deformities can lead to neurovascular compromise of the limb. Hence a 1.5 cm shortening was done through the trapezoidal wedge which prevented any of such complications. The distal femur fragment was then compressed against the shaft and fixed with a distal femur locking plate (Fig. 3). Post-operatively the patient’s lower limbs were immobilized in above knee casts. The casts were removed at 3 weeks and physiotherapy in the form of passive and active movements of the knee joints was performed. The Post-operative Hip & Knee range of motion was 0–90 degrees. The patient achieved standing with the help of calipers and wheelchair mobilization by 6 weeks post-operatively. The severity of spasticity affecting the patient was the cause of a guarded prognosis in this case. However no intervention was carried out on Right shoulder & Left elbow as there was no functional limitation due to the above mentioned joint involvement (Fig. 4). Clinical image at 2 years follow up where patient is able to sit independently and is able to carry out activities of daily living. (Fig. 5)

## Discussion

A crippling complication that occurs in patients with central nervous system trauma in the 2nd to 3rd decade of life, as traumatic brain injury and SCI often occur at this age [4]. The incidence of NHO after traumatic brain injury is reported to be 11–22% [1]. There is a positive correlation between HO and the severity of brain injury and the extent of trauma. In their study, Garland et al. showed that limb spasticity is associated with a higher risk of developing heterotopic ossification [1, 5]. Considering the classic description of the disease, our patient was a male in the 2nd to 3rd decade of life, with a history of traumatic brain injury, diffuse axonal injury with muscles in a state of spasticity, creating an environment just perfect enough to develop heterotopic ossification.

**Figure:**
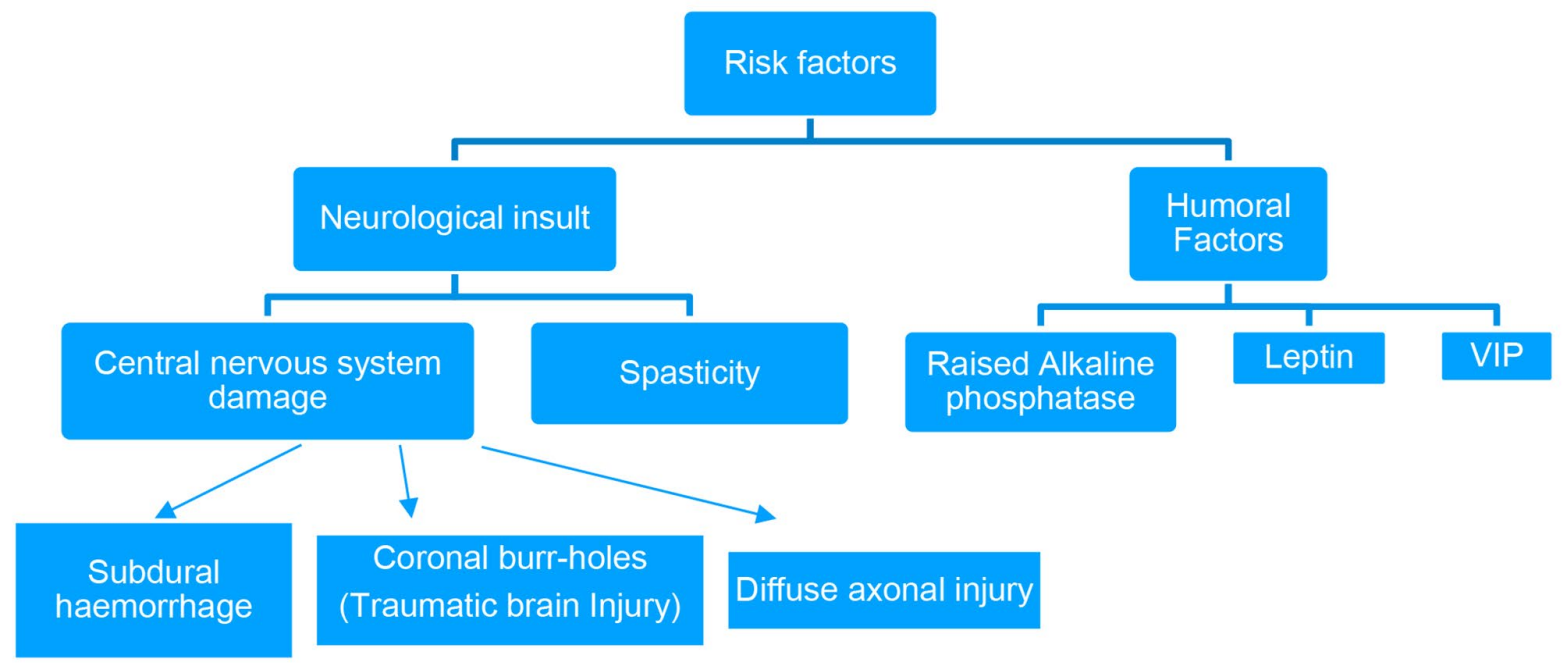

A complex interplay of local and systemic factors, including neuro-endocrine and genetic factors, results in increased osteoblast activity and preferential differentiation of pleuripotent mesenchymal cells into osteoblasts. Serum analysis of patients with traumatic brain and spinal cord injuries has shown the presence of circulating beta factors that can stimulate the differentiation of mesenchymal cells into osteoblasts, which is the basis for heterotopic ossification [4, 6]. In addition, the 24-hour excretion of PGE2 in urine during the initial phase of HO needs to be investigated. Therefore, a PGE2 blocker, indomethacin, is often used to slow down the process of HO in its initial phase [7]. Standard dosing is 75 mg long-acting indomethacin daily, or 25 mg standard release indomethacin three times daily [8].

The clinical presentation of the case usually begins with fever, redness, swelling, and tenderness, which progress to limited range of motion and, in the final stages, ankylosis. The clinical presentation of HO often resembles cellulitis, osteomyelitis or tumor in the early stages.

Heterotopic ossification after head injury usually forms at para-articular sites, with the hip joint being the most common site, followed by the shoulder, elbow, and rarely the knee [On the contrary, the knee joint is the second most common site where heterotopic ossification develops]. Elbow joint is most commonly affected by ankylosis, while it is rare in the knee joint [1].

Devnani A S et al. reported a rare case of a 22-year-old woman who had both hips and knee affected after laparotomy under general anesthesia at the age of 16 years. Excision of heterotopic ossification was performed gradually, and no recurrence occurred for up to 3 years [9].

Xianghong Zhong et al. (2014) published a case report about a 47-year-old man who developed heterotopic ossification in both hips and knees after encephalitis. A holistic approach was considered for treatment, in which the patient was treated surgically in combination with concomitant pharmacotherapy with celecoxib 200 mg for 8 weeks after surgery [10].

Several hematologic (ALP, ESR, CRP, leptins) and radiologic examinations are required to diagnose HO. The average time for diagnosis HO from the onset of the underlying procedure is 2 to 12 months. Serum alkaline phosphatase levels were five times above normal (614 IU/L) at the time of presentation. Serial bone scintigraphy helps monitor the metabolic activity of HO (it becomes positive approximately 3 weeks after the onset of HO) and determine the appropriate timing for surgical resection. Histologically, HO shows a circumferential ossification pattern that appears radiologically as circumferential ossification with a radiolucent centre (becomes positive 4–6 weeks after onset of symptoms). Computed tomographic images help to visualise the anatomy and its extension, which is helpful for preoperative planning [4].

Guidelines for the management of HO have been established based on the maturity of the disease. Comprehensive management is required, including various modalities such as physiotherapy, pharmacotherapy, surgical intervention, and radiation therapy. Our goal was to transition the bedridden patient to a wheelchair to avoid complications associated with diseases of recumbency. Guided physical therapy includes active range of motion exercises, gentle stretching, and resistance exercises ROM without forcible passive manipulation of the joint.

Our patient received indomethacin 75 mg long-acting daily dosage for 6 weeks after surgery and a baclofen tablet 10 mg daily administration tomanage spasticity. Garland et al. (1985) showed that good functional outcomes can be achieved after surgical excision in patients with traumatic brain injury who have intact cognition and adequate motor control [11].

For surgical excision, our plan called for a staged operation with meticulous dissection as these were major surgeries and performing them simultaneously would increase the morbidity significantly. Our goal was to increase the range of motion, so only the ossified nuclei that affected the mobility of joints were removed. The main aim was to achieve a functional resection rather than a complete one, because a complete resection is associated with greater soft tissue trauma, longer operative time, and more bleeding, thus increasing the morbidity of the procedure and the risk of recurrence.

After three surgeries, we were able to provide the patient wheelchair mobilization as well as upright posture which was in line with our goals of managing this case.

## Data Availability

The data and materials has been provided and any additional data that will be required will be provided on timely basis.
